# Epicardium Formation as a Sensor in Toxicology

**DOI:** 10.3390/jdb1020112

**Published:** 2013-07-24

**Authors:** Peter Hofsteen, Jessica Plavicki, Richard E. Peterson, Warren Heideman

**Affiliations:** Department of Pharmaceutical Sciences, University of Wisconsin, 777 Highland Ave, Madison, WI 53705, USA

**Keywords:** epicardium, proepicardium, TCDD, AHR, heart regeneration

## Abstract

Zebrafish (*Danio* rerio) are an excellent vertebrate model for studying heart development, regeneration and cardiotoxicity. Zebrafish embryos exposed during the temporal window of epicardium development to the aryl hydrocarbon receptor (AHR) agonist 2,3,7,8-tetrachlorodibenzo-*p*-dioxin (TCDD) exhibit severe heart malformations. TCDD exposure prevents both proepicardial organ (PE) and epicardium development. Exposure later in development, after the epicardium has formed, does not produce cardiac toxicity. It is not until the adult zebrafish heart is stimulated to regenerate does TCDD again cause detrimental effects. TCDD exposure prior to ventricular resection prevents cardiac regeneration. It is likely that TCDD-induced inhibition of epicardium development and cardiac regeneration occur via a common mechanism. Here, we describe experiments that focus on the epicardium as a target and sensor of zebrafish heart toxicity.

## 1. Introduction

Heart disease remains the leading cause of death worldwide. In the United States, one third of all deaths can be attributed to cardiovascular disease [1]. It is believed that the etiology of heart disease results from a combination of factors including individual genetic makeup and either intentional or unintentional exposure to environmental factors such as tobacco smoke or environmental contaminants at different stages of life. Here, we describe recent work identifying the epicardium as the target for chemicals that cause heart failure in developing embryos.

Although literature searches retrieve hundreds of articles using the keyword epicardium along with toxicity or chemical, the majority of these identify articles that are either about the epicardium as a surface for electrophysiological contact, or articles with the pericardium but no mention of the epicardium. Of the few remaining articles, most examine the epicardium in adults, rather than studies examining epicardium formation or even expansion or maintenance.

It has long been known that drugs used in treating cancer have effects on humans that include damage to the epicardium and other heart structures [2,3]. While not directly related to using the epicardium, it should be noted that the pig pericardium is of intense interest as a biomaterial used in valve reconstruction. Chemical treatment and effects on this pericardial tissue is of interest both as it pertains to human responses to latent material in the xenograft as well as the effectiveness in treatment in prolonging the material effectiveness, primarily reducing calcification [4–6]. Finally among asbestos workers, mesotheliomas grow on various internal tissues. Although primarily associated with the lung, these sometimes arise on the pericardium [7].

Mammalian models have been used to study various aspects of chemical interactions with the epicardium. Iminashi and Arita used ouabain to alter the action potential duration on the epicardium of Japanese monkeys [8]. In another study, the effects of kallikrein was studied on the adult dog epicardium, following the effects of exogenous bradykins and proteases as well as bradykin receptor antagonists on blood pressure and heart rate [9]. Isolated epicardial cells have been used to test the effects of 4-Aminopyridine in ischemia response models [10]. Canine epicardial cells have also been used to test the effects of different adrenergic receptor agonists and antagonists in dogs in the acute stage of Chagas disease [11]. Other studies with dog epicardium examined the effects of captopril on the production of free radicals after electric shock [12].

Others have used the epicardium to study the toxic effects of doxorubicin on conduction properties of the ventricle in the rat [13]. In another study examining mouse epicardial cells, arsenite was found to inhibit the epithelial to mesenchymal transition (EMT) of the epicardial cells, but not differentiation into smooth muscle cells [14]. Using the chick embryo as a model, Olivey *et al* found that transforming growth factor-beta 1 and 2 increase EMT in PE explants [15]. Mouse hearts have also been used as whole explants to follow the effects of antiarrhythmic drugs, often focusing on the excitability of the epicardial layer [16].

There are an increasing number of articles showing the ability of different chemicals to cause pericardial edema in developing fish [17–23]. As will be seen below, the pericardial edema may be a secondary response to the effects of the chemical on epicardium formation. Thus, fish models may prove very sensitive in identifying epicardium-toxic compounds.

Due to the paucity of reports investigating epicardium formation following exposure to environmental chemicals, we describe experiments conducted in our laboratory using zebrafish and mouse models that have identified the epicardium as a target underlying cardiotoxicity. As an example, we use 2,3,7,8-tetrachlorodibenzo-*p*-dioxin (TCDD), a cardiotoxic environmental contaminant and prototypical AHR ligand to show how a common form of cardiotoxicity in zebrafish is connected to impaired epicardium development. We also discuss another cardiotoxic compound and non-AHR agonist, valproic acid (VPA), and its role in zebrafish epicardium development. We speculate that formation of the epicardium may be sensitive to disruption by environmental exposures during vertebrate development.

## 2. Zebrafish Heart and Epicardium Development

The zebrafish heart is made up of two chambers, an atrium and a ventricle. In addition, the bulbous arteriosus lies directly after the ventricle, storing pulse pressure in a role analogous to that of the mammalian aorta. At the inflow tract, the sinus venosus collects venous blood for delivery to the atrium. Blood flows from the sinus venosus through the two contractile chambers and out the bulbous arteriosus to the ventral aorta, which delivers blood to the pharyngeal arch arteries [24].

The vertebrate heart is the first internal organ to form and function [25]. For detailed reviews about early zebrafish heart development see [26,27]. Briefly, in the earliest stages of zebrafish heart development, just prior to gastrulation, bilateral clusters of cardiac progenitor cells are located in the lateral marginal zone. During gastrulation, cardiac progenitors migrate to the anterior lateral plate mesoderm where they are positioned near the midbrain-hindbrain boundary. Myocardial and endocardial precursors converge at the embryonic midline forming a cardiac disc at 19 hours post fertilization (hpf). The cardiac disc undergoes morphogenetic movements to form a contractile linear heart tube (24–28 hpf), which consists of an inner endocardial lining continuous with the vascular endothelium and an overlying myocardial layer. Distinct ventricular and atrial chambers form (30 hpf), the heart tube loops (36 hpf) and the atrioventricular (AV) canal becomes visible. Between the chambers, the endocardium gives rise to endocardial cushions, which are later elaborated into cardiac valves. Proper valve development is essential for directional blood flow and heart function. Continued development of the zebrafish heart is highly dependent on the formation of the third and outer most layer of cardiac tissue, the epicardium.

The epicardium (*epi*: outer, *cardium*: heart) is a mesodermally derived squamous epithelium that is critical for heart development, function and regeneration. Unlike the cells that form the heart tube, the epicardium is derived from a transient pool of progenitors located outside of the heart field, termed the proepicardium (PE) [28–30]. PE cells migrate towards the looped heart and envelop the naked myocardium. In chicks, following initial epicardial coverage of the heart, a subset of epicardial cells undergoes an epithelial-to-mesenchymal transition (EMT) to become epicardial derived progenitor cells (EPDCs; reviewed in [31]. EPDCs invade the underlying myocardium and differentiate directly to become vascular smooth muscle cells and cardiac fibroblasts. Furthermore, EPDCs play important roles in heart looping, valve and coronary vasculature development, cardiac morphogenesis, cardiomyocyte alignment and proliferation, and maturation of the cardiac conduction system (reviewed in [31–33]. Furthermore, it is thought that epicardial cells retain plasticity as cardiac stem cells that can assist adult cardiac regeneration [33–35]. However, the formation or role EPDCs in zebrafish remains undetermined.

## 3. Zebrafish as a Model to Study Cardiotoxicity

Zebrafish are an excellent vertebrate model for studying human health and disease. Development of zebrafish internal organs can be monitored *in vivo* because embryos are translucent and develop externally. These attributes combined with high fecundity and potential for high throughput screening make the zebrafish an ideal model to study heart development and chemical toxicity [27,36]. Zebrafish were first developed as a genetic model, and are highly amenable to forward and reverse genetic screens. These screens have yielded much new information about heart development. Identifying genes required for heart development is greatly facilitated by the fact that zebrafish embryo-larvae are able to rely on passive oxygen diffusion and do not require heart function during the first week of life [37–39]. This is very important because it means that one can follow the effects of mutations interfering with heart function as the heart ceases to function. In mammals, these mutations often produce immediate death of the fetus at the onset of effect.

## 4. DLCs and Heart Defects

Halogenated aromatic hydrocarbons (HAH) are ubiquitous environmental contaminants produced in industrial manufacturing processes and combustion. Included in this class are polychlorinated biphenyls, polychlorinated dibenzofurans, and polychlorinated dibenzo-*p*-dioxins. A subset of these different chemicals shares a similar molecular shape, matching the binding site of the ligand-activated aryl hydrocarbon receptor (AHR). The prototype agonist for the AHR is 2,3,7,8-tetrachlorodibenzo-*p*-dioxin (TCDD), commonly referred to as “dioxin”. Thus, members of this group of AHR agonists can be referred to as dioxin-like compounds (DLCs).

Embryonic exposure to TCDD disrupts cardiac development and function in fish, birds, and mammals [40–45]. TCDD is known to be highly toxic. In humans, epidemiological studies have linked TCDD exposure to congenital heart defects such as hypoplastic left heart syndrome [46,47] and ischemic heart disease [48,49].

The most sensitive organisms to the effects of DLCs are fish, especially fish in early life stages [42,50]. Using zebrafish as a model, it was quickly determined that TCDD exposure during early development causes decreased cardiomyocyte proliferation, a block and lack of erythrocyte development, reduced blood flow and cardiac output, and lack endocardial valve cushions. Ultimately, this leads to ventricular standstill and death [43,51–55]. Understanding why TCDD and related AHR agonists cause cardiotoxicity has been a question for quite some time.

## 5. TCDD and the Zebrafish Epicardium

In zebrafish, the temporal window of cardiotoxicity is peculiar. The first 48 hours of cardiac development proceeds normally when zebrafish are exposed to TCDD immediately following fertilization. The primitive heart tube forms, the heart loops and heart function is indistinguishable from controls. Circulation appears normal at 48 hpf in these animals. After 48 hpf, TCDD treated hearts diverge from experimental controls: the heart unloops and elongates, the ventricle becomes constricted, and pronounced pericardial edema develops [53]. This unusual sensitivity to the cardiotoxic effects of TCDD corresponds to the developmental period in which the PE is specified and migrates over the myocardium to form the epicardium [28,56]. Furthermore, a similar phenotype was observed in the zebrafish *heartstrings* mutant [57]. These fish lack *tbx5a,* which was later shown to be required for PE specification [58].

We now know that TCDD-induced activation of AHR prevents the development of both the PE and epicardium. This results in severe heart malformations that culminate in death [56]. This explains the peculiar lag in toxicity until approximately 48 hpf. Furthermore, after the epicardium has formed it is no longer affected by TCDD; in adults a lethal TCDD exposure fails to produce cardiotoxicity [50,59]. The failure to form an epicardium explains most of the heart malformations caused by TCDD.

Because many other chemicals produce a syndrome including pericardial edema and heart malformations in zebrafish, the epicardium may be useful as a sensor of chemical cardiotoxicity. Furthermore, disruption of epicardial development may prove to be a common cause of cardiotoxicity in developing vertebrates.

## 6. TCDD and the Murine Epicardium

Consistent with the high sensitivity that developing fish have to DLCs, the mouse heart is less sensitive to embryonic TCDD exposure than the zebrafish heart. Unlike zebrafish, TCDD-exposed mice do not exhibit pronounced heart malformations; however, measureable defects such as bradycardia and cardiac hypertrophy have been observed [40]. Additionally, in the fetal murine heart, TCDD-exposure causes global transcriptomic changes in genes that regulate the cell cycle and extracellular matrix [60]. In contrast to the effect in fish, the changes in the mouse heart are not embryonically lethal. Whether they reduce fitness or affect adult health is not known.

**Figure 1:**
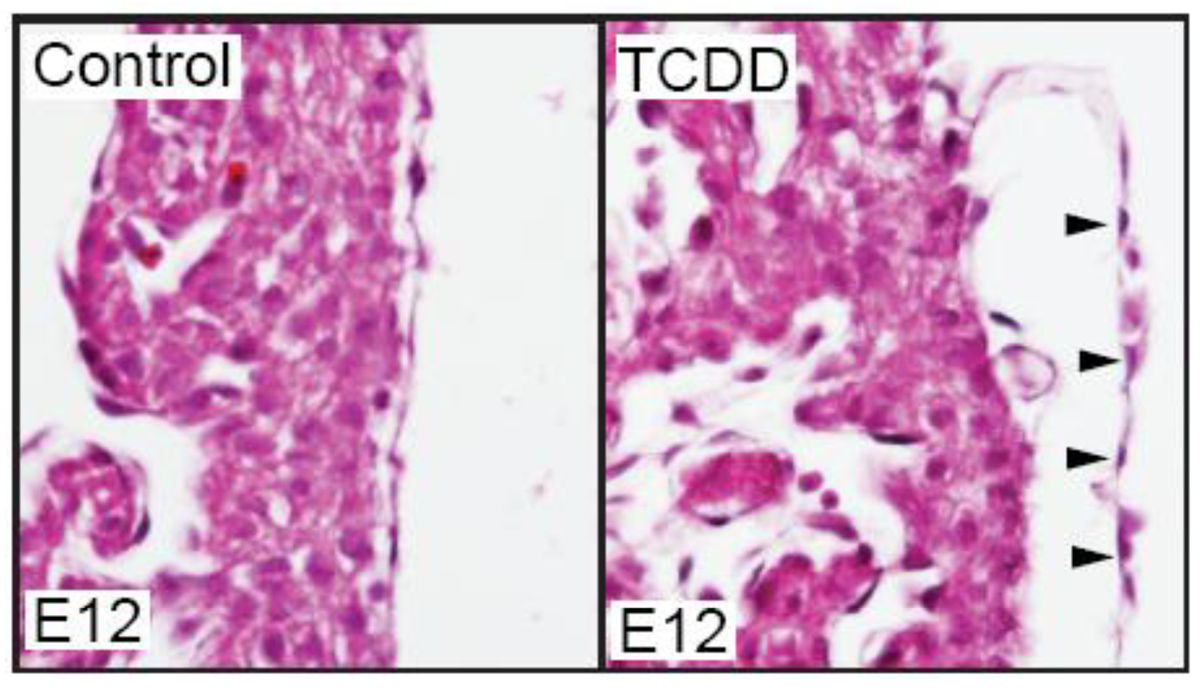
TCDD exposure causes epicardium detachment from the underlying myocardium in developing mice. Murine dams were exposed to vehicle control (corn oil) or TCDD (5 μg/g) at E7 and embryos were collected at E12 for sectional hematoxylin and eosin staining (n = 3/group). Shown are representative sections of control and TCDD hearts at the epicardium. Black arrows denote epicardial cells and their detachment from myocardium.

We have recently found that TCDD exposure does affect the developing mouse epicardium. While TCDD did not prevent epicardium formation in the developing mouse, the epicardium appeared detached from the underlying myocardium (Figure 1). Epicardial detachment has also been observed in mice carrying mutations in RXR-alpha [61], Tcf21 [62], PDGF [63], and Flrt2 [64]. It is generally thought that epicardial detachment in these mutants is due to defects in extracellular matrix composition and EMT processes. Why the epicardium responses differ between mouse and zebrafish is an intriguing question for further investigation.

## 7. The Zebrafish Epicardium as a Sentinel for Other Cardiotoxic Compounds

**Figure 2:**
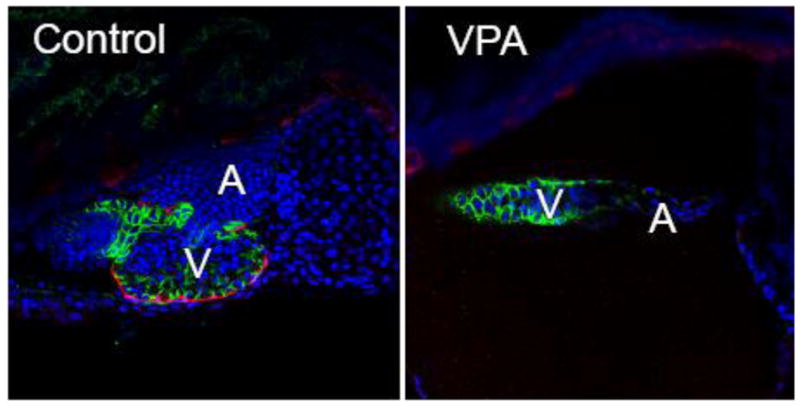
VPA exposed zebrafish larvae lack epicardium. Embryos from a zebrafish epicardial reporter line (*tcf21:DsRed*; [83] were exposed to VPA (0.06 μM), or vehicle (EtOH) shortly following fertilization. The control is shown at left, while the VPA heart is shown at right. The presence or absence of epicardium was assessed with immunohistochemistry at 120 hours post fertilization using an antibody against *tcf21*, expressed in epicardial cells (Red). Cell nuclei are stained with DAPI shown as blue. Cytoplasmic *cmlc2:GFP* shown as green outlines the cardiomyocytes. Representative single plane confocal microscopy images (n = 6/group) are shown with anterior to the left (V = ventricle, A = atrium).

Pericardial edema coupled with an elongated, unlooped heart is a phenotype that is not unique to fish exposed to DLCs. This syndrome has been observed in zebrafish carrying mutations that disrupt the cardiovascular and other systems, as well as following exposure to a variety of cardiotoxic chemicals [65–67]. Based on our results with TCDD, a possible link between these factors and the syndrome of heart malformation and pericardial edema could be effects on epicardium formation. We have made a preliminary test of this idea using valproic acid (VPA), a compound that is not a DLC, yet produces the type of cardiac malformations described for TCDD. VPA is an anticonvulsant known to affect cardiovascular development in zebrafish [68]. Despite critical differences in the mechanism of action, both agents produced an elongated heart with a thin atrium, a compacted ventricle, and failure of epicardium development (Figure 2).

Of particular interest, VPA-exposed zebrafish appeared completely normal until the PE to epicardium transition, in which the cluster of cells forming the PE begins to cross over to the heart to form the epicardium. In VPA-treated zebrafish, there were no signs of pericardial edema during the period from 0–72 hpf. While the PE formed in these fish, there was no progression to forming the epicardium. In the ensuing period from ~72–120 hpf, during which the epicardial cells would normally envelop the heart, the hearts in the VPA-treated zebrafish became unlooped and elongated. This was accompanied by marked pericardial edema.

While the mechanism remains a mystery, epicardium formation was sensitive to blockade by two compounds with very distinct mechanisms of action. In both cases, the initial patterning of the heart was unaffected, and cardiotoxicity was only manifested at the point at which epicardial cells normally form. It may be that epicardium formation is more sensitive to disruption than other processes needed to produce a healthy functional heart. If so, the epicardium could be a sentinel for cardiotoxicity.

## 8. The Epicardium and Heart Regeneration

Following myocardial infarction, the human heart can lose billions of cardiomyocytes, resulting in the loss of a significant portion of myocardium [69]. The damaged myocardium is not replaced with new myocardial cells, but rather healed by formation of non-contractile scar tissue. This inability to replace lost heart tissue with contractile myocardium is a critical medical problem [70,71].

The epicardium has received great interest in the field of regenerative medicine due to the plasticity of epicardial progenitors (reviewed in [32–35,72]. Furthermore, many reports have identified the epicardium as a critical signaling center following myocardial injury.

Unlike mammals, zebrafish retain the ability to regenerate damaged myocardial tissue into adulthood [73]. This occurs mainly by differentiation and proliferation of spared resident cardiomyocytes [74,75]. Following surgical resection of the adult zebrafish ventricle at its apex, profuse bleeding occurs followed by the formation of a fibrin-rich blood clot. Subsequently, an epicardial-derived white epithelial sheath of tissue migrates and surrounds the newly formed blood clot [76]. The combination of sealing the wound with epithelial-like sheath of tissue, cardiomyocyte dedifferentiation and proliferation orchestrate regeneration of a new contractile myocardium over a period of 1–2 months.

During heart regeneration in zebrafish, the epicardium is thought to revert to an “embroyonic-like” state and act as a signaling center by expressing embryonic genes such as the retinoic acid (RA) synthesizing enzyme, *raldh2* [76,77]. Expression of *raldh2* in epicardial and endocardial cells during regeneration is thought to secrete trophic RA to assist in cardiomyocyte proliferation. Inhibition of RA signaling during heart regeneration reversed the regenerative capacity by halting cardiomyocyte proliferation [78]. While controversy remains regarding the role of the epicardium during heart regeneration, it appears the adult epicardium may play a supportive role by acting as a signaling center to direct differentiation, proliferation and recruitment of cardiac progenitors to the wound site. Thus, embryonic-like epicardial cells may be needed for wound healing in the adult heart.

Once the epicardium has formed, the heart appears to no longer be a target for DLCs in zebrafish, and even when exposed to a lethal concentration of TCDD the adult heart is unaffected [50,59]. However, as indicated above, embryonic-like epicardial progenitor cells are required for heart wound healing in the adult zebrafish. It is perhaps then not surprising that during wounding, the adult zebrafish regains sensitivity to TCDD. In experiments in which the apex of the adult ventricle were amputated, control hearts quickly regenerated the missing tissue, while TCDD-pretreated hearts did not progress past the initial stage of blood clot formation [59]. TCDD prevented the formation of the epicardially-derived sheath of tissue that normally envelops the wound, and reduced cardiomyocyte proliferation at the wound site. If TCDD was administered one day following surgical amputation, the regenerative capacity was not affected; the epithelial-like sheath of tissue formed and the blood clot was replaced with new contractile myocardium. This suggests that activated epicardial progenitors in the adult partially amputated zebrafish heart are targeted by TCDD.

## 9. Conclusions

The epicardium and epicardial progenitors are potentially vital for development, function and regeneration of the vertebrate heart. In zebrafish, the epicardium appears to be sensitive to disruption by xenobiotic chemicals during development, but not once formed. In humans, long-term exposure to TCDD is associated with ischemic heart disease [48,49]. It is interesting that other studies of ischemic heart disease have found a reduction in epicardial cell numbers in affected patients [48,49,79]. We speculate that epicardium development may play an important role in environmentally induced heart disease, worth further study. The zebrafish heart is now being used to screen drug candidates for Q-T prolongation and other forms of cardiotoxicity [80–82]. Due to high throughput screening capability, zebrafish have many advantages in screening for teratogenic effects over other vertebrate models. It may be that the tools available with the zebrafish—transgenic lines that mark the epicardium, translucent external development, and high fecundity—will make following epicardium development an important screen as well. Thus, we may see future use for epicardium formation as a marker of cardiotoxicity.
